# PATTERNS OF AGING CHANGES IN BODY WEIGHT AND BMI MAY PREDICT CHANCES OF ALZHEIMER'S DISEASE AND LONGEVITY

**DOI:** 10.1093/geroni/igac059.1287

**Published:** 2022-12-20

**Authors:** Svetlana Ukraintseva, Konstantin Arbeev, Hongzhe Duan, Rachel Holmes, Igor Akushevich, Arseniy Yashkin, Heather Whitson, Anatoliy Yashin

**Affiliations:** Duke University, Durham, North Carolina, United States; Duke University, Durham, North Carolina, United States; Duke University, Durham, North Carolina, United States; Duke University, Durham, North Carolina, United States; Duke University, Durham, North Carolina, United States; Duke University, Durham, North Carolina, United States; Duke University, Durham, North Carolina, United States; Duke University, Durham, North Carolina, United States

## Abstract

**Background:**

Lower bodyweight/BMI was previously linked to AD and frailty; however, the role of long-term changes in the bodyweight/BMI in both AD and longevity is not well understood, as is the role of APOE polymorphism in such changes.

**Methods:**

Using longitudinal data from the Framingham Heart Study (FHS) and the Health and Retirement Study (HRS), we estimated trajectories of the weight and BMI at ages 40 to 75, and compared them between individuals who did and who did not develop AD at ages 75+. We also evaluated associations between APOE4 carrier status and key characteristics of the age-trajectories of weight/BMI, including the age at peak value of the bodyweight/BMI (AgeMax), and slope of the decline in bodyweight/BMI after reaching the maximum.

**Results:**

Women with late-onset-AD had lower bodyweight/BMI values up to three decades before AD diagnosis. They reached the peak of bodyweight in their 50s, about 10 years earlier than AD-free women. Younger AgeMax was associated with lower survival chances after age 80 in women. APOE4 carriers showed earlier/faster declines in weight and BMI than non-carriers; however, relevance of this to AD was unclear.

**Conclusion:**

Younger age at peak value of the bodyweight/BMI indicates higher chances of late-onset-AD, while older age can predict better survival later in life and may favor longevity in women. The earlier start of the decline in bodyweight/BMI values could be sign of accelerated aging, which may contribute to AD. Relevance of APOE4 effects on age-trajectories of weight/BMI to AD warrants further investigation.

